# A Review of Ongoing Trials of Stereotactic Ablative Radiotherapy for Oligometastatic Cancers: Where Will the Evidence Lead?

**DOI:** 10.3389/fonc.2019.00543

**Published:** 2019-06-21

**Authors:** Faiez Al-Shafa, Andrew J. Arifin, George B. Rodrigues, David A. Palma, Alexander V. Louie

**Affiliations:** ^1^Division of Radiation Oncology, London Regional Cancer Program, London, ON, Canada; ^2^Department of Radiation Oncology, Odette Cancer Centre, Sunnybrook Health Sciences Centre, Toronto, ON, Canada

**Keywords:** stereotactic, radiotherapy, SBRT, SABR, oligometastasis

## Abstract

**Purpose:** The oligometastatic state is a proposed entity between localized cancer and widely metastatic disease, comprising an intermediate subset of metastatic cancer patients. Most data to support locally-directed treatment, such as stereotactic ablative radiotherapy (SABR), for oligometastases are from retrospective institutional reports. Following the success of a recently completed and reported phase II trial demonstrating important clinical outcomes, herein we review the current landscape of ongoing clinical trials in this context.

**Materials and methods:** A review of currently activated and registered clinical trials was performed using the clinicaltrials.gov database from inception to February 2019. A search of actively recruiting trials, using the key words oligometastases, SABR, and various related terms was performed. Search results were independently reviewed by two investigators, with discrepancies settled by a third. Data abstracted from identified studies included study type, primary disease site, oncologic endpoints, and inclusion/exclusion criteria.

**Results:** Of the initial 216 entries identified, 64 met our review eligibility criteria after full-text review. The most common study type was a phase II clinical trial (*n* = 35, 55%) with other study designs ranging from observational registry trials to phase III randomized controlled trials (RCTs). A minority of trials were randomized in design (*n* = 17, 27%). While most studies allowed for metastases from multiple primary disease sites (*n* = 22, 34%), the most common was prostate (*n* = 13, 15%), followed by breast, gastrointestinal, non-small cell lung cancer (NSCLC), and renal (*n* = 6, 9% each). In studies with a solitary target site, the most common was liver (*n* = 6, 9%) followed by lung (*n* = 3, 5%). The most common primary endpoints were progression-free survival (PFS) (*n* = 20, 31%) and toxicity (*n* = 10, 16%). A combined strategy of systemic therapy and SABR was an emerging theme (*n* = 23, 36%), with more recent studies specifically evaluating SABR and immunotherapy (*n* = 9, 14%).

**Conclusion:** The safety and efficacy of SABR as oligometastasis-directed treatment is increasingly being evaluated within prospective clinical trials. These data are awaited to compliment the abundance of existing observational studies and to guide clinical decision-making.

## Introduction

Metastatic cancer is a heterogeneous entity on a spectrum that ranges from a single metastasis to widely disseminated disease. Historically, patients with metastatic disease were generally considered incurable whereby palliative systemic therapy is the primary treatment and radiotherapy is reserved for palliation of symptoms (1). Today, the concept of oligometastases has diffused into the medical vernacular, and it represents an intermediate state between locoregionally confined cancer and widespread metastases whereby the number of metastases and organs are limited, typically between 1 and 5 lesions. By nature of having limited spread, it has been postulated that with aggressive metastasis-directed therapy, one can achieve better than expected survival, and in some scenarios, cure (2).

The oligometastatic state can also be further defined by its chronicity and evolvement. Synchronous oligometastatic disease is defined as *de novo* presentation of a primary cancer associated with limited metastases. In contrast, metachronous oligometastatic disease refers to the development of a few metastases after a primary cancer is detected. The term oligo-recurrence describes the development of metachronous oligometastases with a controlled primary site (3). Meanwhile, oligoprogression describes a state in which a limited number of metastatic lesions progress, while all other sites of disease remain stable, typically while on systemic therapy (4, 5). As each of these definitions represents a distinct scenario with a range of associated prognoses, classification of the appropriate type of oligometastasis is crucial both in the clinic and when appraising the growing outcomes-based literature.

The clinical implication of oligometastatic state is that cure or long-term survival can be achieved for this subset of patients with metastatic disease. Initially, reports on favorable survival outcomes in oligometastatic cancers largely involved surgery (6). In 1997, the International Registry of Lung Metastases reported a 5-year overall survival (OS) of 36% in patients with lung metastases treated by surgical resection (7). Moreover, a 5-year OS of 40% was reported following liver resection for metastatic colorectal cancer patients with a median survival of 46 months (8). A retrospective chart review from a single institution reported a 5-year OS of 70% among 12 patients with non-small cell lung cancer (NSCLC) after complete surgical resection of synchronous or metachronous brain metastases followed by whole brain irradiation (9). A review of 10 articles examining the outcomes of adrenalectomy for isolated synchronous and metachronous adrenal metastases in NSCLC reported a 5-year OS of 25% (10).

Currently, stereotactic radiosurgery (SRS) is generally considered to be the recommended treatment option for resected cavity and non-resected brain metastases (11). In a retrospective study involving 42 patients with synchronous solitary brain metastases from NSCLC, a 5-year OS of 21% was reported (12). Consequently, the use of metastases directed ablative therapy in the form of stereotactic ablative radiotherapy (SABR) has rapidly increased. SABR is a modern radiation technique that achieves highly accurate targeting, very conformal dose distributions and delivers highly ablative dose over a short overall treatment duration, usually in 1–5 treatments. A systematic review reported a 2-year local control rate of 77.9% and a 2-year OS of 53.7% for patients with lung oligometastases treated with SABR (13).

The clinical evidence to support SABR as a minimally invasive treatment for oligometastatic disease comprises of, in decreasing order of abundance, single-institution retrospective series, multi-institutional retrospective series, single-arm prospective trials, and randomized controlled trials (RCTs). Several RCTs have been published thus far.

The first was a phase II multicenter trial examined local consolidative therapy (LCT), including surgery or SABR, vs. maintenance therapy or observation for patients with oligometastatic NSCLC without progression after first-line systemic therapy (14). The study was closed early when interim analysis demonstrated a significantly longer median progression-free survival (PFS) in the LCT group vs. maintenance therapy group, 11.9 vs. 3.9 months, respectively (HR 0.35; 90% CI: 0.18–0.66; *p* = 0.0054). At final analysis, median OS was also significantly longer in patients in the LCT group than in the maintenance treatment group, 41.2 vs. 17.0 months, respectively (*p* = 0.017), with no additional grade III or higher toxicity. Similarly, a recent phase II single center RCT examined maintenance chemotherapy with or without LCT following partial or complete response on first-line platinum-based induction chemotherapy for NSCLC (15). This study was also closed early as PFS was nearly triple in the LCT arm vs. maintenance chemotherapy arm alone (9.7 vs. 3.5 months, respectively; *p* = 0.01). There was no difference in toxicity between the arms. Median OS was not reached in the SABR-maintenance chemotherapy arm, though the study was not powered to show a statistical difference in this measure.

The STOMP trial examined the effect of metastasis-directed therapy for oligometastatic prostate cancer (16). It was a phase II multicenter RCT that compared LCT vs. surveillance with oligometastatic prostate cancer detected on choline positron emission tomography–computed tomography. The authors found that androgen-deprivation therapy-free survival was higher in the LCT arm compared to the surveillance arm, 21 vs. 13 months, respectively (HR 0.60; 80% CI: 0.40–0.90; *p* = 0.11). No grade 2 or higher toxicity was observed.

Our group recently published the results from SABR-COMET, a phase II multicenter RCT for metachronous oligometastases of any origin (17). The study compared SABR vs. standard of care palliative treatment for up to 5 metastatic lesions among 99 patients. Median OS was 28 months for the standard of care treatment arm vs. 41 months in the standard of care treatment plus SABR arm (HR 0.57; 95% CI 0.30–1.10; *p* = 0.090). SABR was well-tolerated with no difference in overall quality of life at 6 months (*p* = 0.99). There were three (4.5%) treatment-related deaths in the SABR arm.

There is an increasing worldwide trend toward the use of SABR for oligometastatic cancers, despite a paucity of prospective data to support this strategy (18). Nonetheless, a number of clinical trials have been designed and are actively accruing. In this review, we aim to summarize the current state of registered oligometastatic clinical trials using SABR for oligometastasis-directed treatment.

## Materials and Methods

Clinicaltrials.gov is a database registry of privately and publicly funded clinical studies worldwide. A search was performed in the clinicaltrials.gov registry from inception to February 19, 2019. A combination of search terms was used to capture trials that reported on SABR (“stereotactic,” “stereotaxis”) for metastases (“oligo-, metastatic, metastasis, metastasize”). All trials underwent full text review by two independent reviewers. A third reviewer was available in case of a discrepancy between the two initial reviewers. Inclusion criteria included:

- Population: trials with inclusion criteria that limited the number of metastases throughout the whole body to any upper limit. This ranged from 3 to 10 metastases. Metastases were allowed from any primary disease site.
- Intervention: at least a proportion of the study population must undergo SABR. This can be combined with other local therapies (surgery, radiofrequency ablation), and/or systemic therapies.
- Recruitment status: actively recruiting.

Data abstracted from selected studies included study design, primary disease site(s), target site(s), population, outcomes, and inclusion/exclusion criteria.

## Results

**Figure 1:**
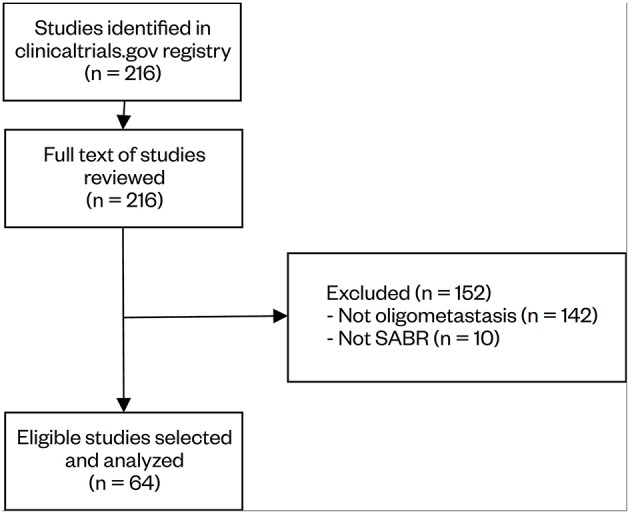
Selection process of clinical trials regarding oligometastasis-directed SABR treatment. The initial search identified 216 studies, which were reviewed by two independent reviewers. Based on the eligibility criteria, 64 studies were selected for analysis.

The initial search identified 216 studies. The study description, recruitment status, and inclusion/exclusion criteria were reviewed for relevance. In total, 64 studies were selected for data collection. Of the 152 excluded studies, reasons for exclusion included not limiting the number of metastases (*n* = 142), and not having SABR as an intervention (*n* = 10). Notably, a large number of brain and spine SABR trials defined oligometastases in a solitary target site and did not limit the number of metastases elsewhere in the body, hence its exclusion from this review. The study selection process is summarized in Figure 1.

Details of the reviewed studies are summarized in Table 1. The most common study type was a phase II clinical trial (*n* = 35, 55%) with other study designs ranging from observational registry trials to phase III RCTs. A minority of trials were randomized in design (*n* = 17, 27%). While most studies allowed for metastases from multiple primary disease sites (*n* = 22, 34%), the most common was prostate (*n* = 13, 15%), followed by breast, gastrointestinal, NSCLC and renal (*n* = 6, 9% each). In studies with a solitary target site, the most common was liver (*n* = 6, 9%) followed by lung (*n* = 3, 5%). Of note, there was 1 trial (2%) targeting the pediatric population. The most common primary endpoints were PFS (*n* = 20, 31%) and toxicity (*n* = 10, 16%). A combined strategy of systemic therapy and SABR was an emerging theme (*n* = 23, 36%), with more recent studies specifically evaluating SABR and immunotherapy (*n* = 9, 14%).

**Table 1:** Summary statistics of the analyzed studies.

**Study phase**	
Phase I	4 (6%)
Phase I/II	7 (11%)
Phase II	35 (55%)
Phase II/III	5 (8%)
Phase III	5 (8%)
Observational studies	4 (6%)
Unspecified	4 (6%)
**Primary disease site**	
Breast	6 (9%)
Gastrointestinal	6 (9%)
Head and neck	2 (3%)
Non-small cell lung cancer	6 (9%)
Prostate	13 (20%)
Renal	6 (9%)
Multiple	22 (34%)
**Target site**	
Brain	2 (3%)
Liver	6 (9%)
Lung	3 (5%)
Spine	1 (2%)
Unspecified/multiple	52 (81%)
**Population**	
Pediatrics	1 (2%)
**Primary endpoint**	
Dose, planning	6 (9%)
Feasibility	3 (5%)
Overall survival	5 (8%)
Progression-free survival	20 (31%)
Toxicity	10 (16%)
**Study design**	
Non-randomized comparison	5 (8%)
Randomized	17 (27%)
Single treatment	38 (59%)
**Disease state**	
Metachronous metastases only	7 (11%)
Synchronous metastases only	5 (8%)
Oligoprogression only	6 (9%)
Oligorecurrence only	3 (5%)
Neither oligoprogression or oligorecurrence (*de novo* only)	5 (8%)
**Systemic treatments**	
Prior systemic treatments allowed	26 (41%)
Trial combines SABR with systemic treatment	23 (36%)
Trial combines SABR with immunotherapy	9 (14%)

## Discussion

This review of active clinical trials evaluating the use of SABR in the setting of oligometastases illustrates that significant prospective efforts are underway to help inform decision-making in various scenarios. Although the number of trials identified is encouraging, there are a number of caveats. First, a lack of consistency in the definition of the type and number of oligometastases studied may limit the generalizability of these trials. Second, few were randomized in design, and many had non-definitive endpoints such as PFS or toxicity. Further, many trials combined SABR with other local treatments and/or systemic therapies, which presents challenges in measuring the direct risks and benefits of SABR. Finally, many trials employing brain and spine SABR in a solitary target site were excluded from analysis, as they included both oligometastatic and polymetastatic patients. Thus, this overview may not fully address the relative merits of SABR in central nervous system targets.

A recent literature review highlighted the importance of differentiating among the subtypes of oligometastatic states. An analysis of 17 publications comprising 869 patients who underwent SABR for lung oligometastases demonstrated that the cohort of patients with a disease-free interval of longer than 24 months conferred higher OS than those without (19). This supports the theory that there is a prognostic difference between those with synchronous and metachronous oligometastasis and raises the possibility of a difference between those with oligo-recurrence and oligoprogression.

In the absence of abundant prospective clinical trial data, there have been various epidemiological studies to help guide prognosis. For example, the METABANK score is a predictive nomogram for survival after stereotactic radiotherapy for oligometastatic disease based on a retrospective analysis of 403 patients who received SABR for 1–5 metastatic sites at a single institution (20). Three parameters had a high independent impact on survival: presence of brain metastases, non-adenocarcinoma histology, and low performance score.

A multi-institutional pooled analysis of 361 patients with extracranial oligometastatic disease who received ablative doses of radiotherapy found that prognostic factors associated with higher OS included age, number of metastases, primary tumor type, time to metastatic diagnosis, metastatic site, and a biological equivalent dose of ≥75 Gy (21). Another pooled analysis of 700 patients with lung metastases treated with SABR reported better outcomes for patients with good performance status, single vs. multiple pulmonary metastases, breast or colorectal primary vs. NSCLC and sarcoma, and a longer time interval between the initial primary tumor diagnosis and the SABR treatment (22). Further work is needed to further characterize the biological basis behind these prognostic indicators.

There is a growing interest in the role of SABR in anti-cancer immunity, as evidenced by the number of trials combining SABR and immunotherapy and using the abscopal effect as a secondary endpoint. The abscopal effect describes the theoretical ability of localized radiation inducing regression and response of non-irradiated metastatic sites due to a systemic anti-tumor immune response (23). Originally described in multiple case reports in the 1950s, this effect has renewed attention given the recent success of immunotherapies. For example, a subgroup analysis of the KEYNOTE-001 trial of 98 patients with advanced NSCLC treated with pembrolizumab reported that PFS and OS were higher in patients who were previously treated with radiation therapy compared to those without, suggesting a synergistic effect between radiation and immunotherapy (24). This was further suggested in a phase I study that evaluated the safety of pembrolizumab combined with SABR in patients with advanced solid tumors (25). Patients with metastatic disease and progressing on standard treatments received SABR to multiple sites followed by pembrolizumab within 7 days of completing radiation treatment. The authors reported comparable rates of toxicity of SABR or pembrolizumab monotherapy, and tumor control in 36 of 52 (69.2%) patients. In patients who had SABR to multiple but not all metastases, the authors observed a 26.9% response rate in non-irradiated sites.

Additionally, PEMBRO-RT is a phase II RCT examining the effects of pembrolizumab alone vs. SABR followed by pembrolizumab in patients with metastatic NSCLC (26). A recent interim analysis demonstrated a significant increase in median PFS from 1.8 to 6.4 months in the monotherapy and combination therapy arms, respectively (HR 0.55; CI 0.31–0.98; *p* = 0.04). Further, the authors did not observe any significant differences in toxicity between the arms. Meanwhile, a phase II RCT examining the abscopal effect in patients with metastatic head and neck squamous cell cancer by comparing nivolumab alone vs. nivolumab with SABR to a single lesion did not demonstrate a difference in PFS or OS between the two arms (27). It is clear that more clinical trial data is needed to clarify the role of SABR and immunotherapy.

## Conclusion

The safety and efficacy of SABR as oligometastasis-directed treatment is increasingly being evaluated within prospective clinical trials. Emerging themes include differentiating among the subtypes of oligometastatic states and combining SABR and systemic therapies. These data are awaited to compliment the abundance of existing observational studies to guide clinical decision-making. Enrolling in prospective trials evaluating SABR in various clinical scenarios has several benefits beyond the generation of higher quality evidence. Firstly, vigorous quality assurance within trials provides a mechanism to improve the framework of technical nuances within centers that are looking to expand the scope to organ systems not previously treated within the team in a controlled manner. Secondly, the implementation of protocols for trial patients inherently benefits patients who are treated off trial in the same institution by nature of these implementations. Finally, subset analyses of prospective trials for endpoints such as safety can be performed using dosimetric information, which will be invaluable to further refine organ at risk constraints. Ultimately, as the landscape of advanced cancer management rapidly evolves with the rise of immunotherapy, targeted therapies, and other novel agents, clarity on how SABR fits within the proven and purported benefits of these treatments will be a priority area of research moving forward.

## Author Contributions

AL and FA-S contributed to the conception and design of the study. AA organized the database. FA-S wrote the first draft of the manuscript. AA and AL wrote sections of the manuscript. All authors contributed to manuscript revision, read and approved the submitted version.

### Conflict of Interest Statement

AL has received honoraria from Varian Medical Systems Inc. and AstraZeneca. The remaining authors declare that the research was conducted in the absence of any commercial or financial relationships that could be construed as a potential conflict of interest.

## References

[1] Huang F Wu G Yang K. Oligometastasis and oligo-recurrence: more than a mirage. Radiat Oncol. (2014) 9:230. 10.1186/s13014-014-0230-6 25359216 PMC4222373

[2] Palma DA Louie AV Rodrigues GB. New strategies in stereotactic radiotherapy for oligometastases. Clin Cancer Res. (2015) 21:5198–204. 10.1158/1078-0432.CCR-15-0822 26626571

[3] Niibe Y Hayakawa K. Oligometastases and oligo-recurrence: the new era of cancer therapy. Jpn J Clin Oncol. (2010) 40:107–11. 10.1093/jjco/hyp167 20047860 PMC2813545

[4] Correa RJM Salama JK Milano MT Palma DA. Stereotactic body radiotherapy for oligometastasis opportunities for biology to guide clinical management. Cancer J. (2016) 22:247–56. 10.1097/PPO.0000000000000202 27441744

[5] Reyes DK Pienta KJ. The biology and treatment of oligometastatic cancer. Oncotarget. (2015) 6:8491–524. 10.18632/oncotarget.3455 25940699 PMC4496163

[6] Crisci R Barone M Zaccagna G Gabriele F Divisi D. Surgical approach in the oligometastatic patient. Ann Transl Med. (2018) 6:94. 10.21037/atm.2018.01.19 29666817 PMC5890044

[7] Pastorino U Buyse M Friedel G Ginsberg RJ Girard P Goldstraw P. Long-term results of lung metastasectomy: prognostic analyses based on 5206 cases. J Thorac Cardiovasc Surg. (1997) 113:37–49. 10.1016/S0022-5223(97)70397-0 9011700

[8] Choti MA Sitzmann JV Tiburi MF Sumetchotimetha W Rangsin R Schulick RD. Trends in long-term survival following liver resection for hepatic colorectal metastases. Ann Surg. (2002) 235:759–66. 10.1097/00000658-200206000-00002 12035031 PMC1422504

[9] Daniels M Wright GM. Complete resection of non-small-cell lung cancer and oligo-metastatic brain disease. ANZ J Surg. (2005) 75:963–6. 10.1111/j.1445-2197.2005.03585.x 16336388

[10] Tanvetyanon T Robinson LA Schell MJ Strong VE Kapoor R Coit DG. Outcomes of adrenalectomy for isolated synchronous versus metachronous adrenal metastases in non-small-cell lung cancer: a systematic review and pooled analysis. J Clin Oncol. (2008) 26:1142–7. 10.1200/JCO.2007.14.2091 18309950

[11] National Comprehensive Cancer Network *Central Nervous System Cancers* (Version 2.2018) (2018). Available online at: https://www.nccn.org/professionals/physician_gls/pdf/cns.pdf (accessed February 21, 2019).

[12] Flannery TW Suntharalingam M Regine WF Chin LS Krasna MJ Shehata MK. Long-term survival in patients with synchronous, solitary brain metastasis from non-small-cell lung cancer treated with radiosurgery. Int J Radiat Oncol Biol Phys. (2008) 72:19–23. 10.1016/j.ijrobp.2007.12.031 18280058

[13] Ashworth A Rodrigues G Boldt G Palma D. Is there an oligometastatic state in non-small cell lung cancer? A systematic review of the literature. Lung Cancer. (2013) 82:197–203. 10.1016/j.lungcan.2013.07.026 24051084

[14] Gomez DR Blumenschein GR Lee JJ Hernandez M Ye R Camidge DR. Local consolidative therapy versus maintenance therapy or observation for patients with oligometastatic non-small-cell lung cancer without progression after first-line systemic therapy: a multicentre, randomised, controlled, phase 2 study. Lancet Oncol. (2016) 17:1672–82. 10.1016/S1470-2045(16)30532-0 27789196 PMC5143183

[15] Iyengar P Wardak Z Gerber DE Tumati V Ahn C Hughes RS. Consolidative radiotherapy for limited metastatic non-small-cell lung cancer: a phase 2 randomized clinical trial. JAMA Oncol. (2018) 4:e173501. 10.1001/jamaoncol.2017.3501 28973074 PMC5833648

[16] Ost P Reynders D Decaestecker K Fonteyne V Lumen N De Bruycker A Surveillance or metastasis-directed therapy for oligometastatic prostate cancer recurrence: a prospective, randomized, multicenter phase II trial. J Clin Oncol. (2018) 36:446–53. 10.1200/JCO.2017.75.4853 29240541

[17] Palma DA Olson R Harrow S Gaede S Louie AV Haasbeek C. Stereotactic ablative radiotherapy versus standard of care palliative treatment in patients with oligometastatic cancers (SABR-COMET): a randomised, phase 2, open-label trial. Lancet. (2019) 393:2051–8. 10.1016/S0140-6736(18)32487-5 30982687

[18] Lewis SL Porceddu S Nakamura N Palma DA Lo SS Hoskin P. Definitive stereotactic body radiotherapy (SBRT) for extracranial oligometastases: an international survey of >1000 radiation oncologists. Am J Clin Oncol. (2017) 40:418–22. 10.1097/COC.0000000000000169 25647831

[19] Alongi F Mazzola R Figlia V Guckenberger M. Stereotactic body radiotherapy for lung oligometastases: literature review according to PICO criteria. Tumori. (2018) 104:148–56. 10.1177/0300891618766820 29714665

[20] Van den Begin R Engels B Collen C de Vin T Defauw A Dubaere E The METABANK score: a clinical tool to predict survival after stereotactic radiotherapy for oligometastatic disease. Radiother Oncol. (2019) 133:113–9. 10.1016/j.radonc.2019.01.001 30935566

[21] Hong JC Ayala-Peacock DN Lee J Blackstock AW Okunieff P Sung MW. Classification for long-term survival in oligometastatic patients treated with ablative radiotherapy: a multi-institutional pooled analysis. PLoS ONE. (2018) 13:e0195149. 10.1371/journal.pone.0195149 29649281 PMC5896920

[22] Wong AC Watson SP Pitroda SP Son CH Das LC Stack ME. Clinical and molecular markers of long-term survival after oligometastasis-directed stereotactic body radiotherapy (SBRT). Cancer. (2016) 122:2242–50. 10.1002/cncr.30058 27206146

[23] Hu ZI McArthur HL Ho AY. The abscopal effect of radiation therapy: what is it and how can we use it in breast cancer? Curr Breast Cancer Rep. (2017) 9:45–51. 10.1007/s12609-017-0234-y 28344743 PMC5346418

[24] Shaverdian N Lisberg AE Bornazyan K Veruttipong D Goldman JW Formenti SC. Previous radiotherapy and the clinical activity and toxicity of pembrolizumab in the treatment of non-small-cell lung cancer: a secondary analysis of the KEYNOTE-001 phase 1 trial. Lancet Oncol. (2017) 18:895–903. 10.1016/S1470-2045(17)30380-7 28551359 PMC5538772

[25] Luke JJ Lemons JM Karrison TG Pitroda SP Melotek JM Zha Y. Safety and clinical activity of pembrolizumab and multisite stereotactic body radiotherapy in patients with advanced solid tumors. J Clin Oncol. (2018) 36:1611–8. 10.1200/JCO.2017.76.2229 29437535 PMC5978468

[26] Theelen W H.Peulen@Nki.Nl NF Lalezari F de Vries J De Langen J Aerts J Randomized phase II study of pembrolizumab after stereotactic body radiotherapy (SBRT) versus pembrolizumab alone in patients with advanced non-small cell lung cancer: the PEMBRO-RT study. J Clin Oncol. (2018) 36 (15_suppl):9023. 10.1200/JCO.2018.36.15_suppl.9023

[27] McBride SM Sherman EJ Tsai CJ Baxi SS Aghalar J Eng J A phase II randomized trial of nivolumab with stereotactic body radiotherapy (SBRT) versus nivolumab alone in metastatic (M1) head and neck squamous cell carcinoma (HNSCC). J Clin Oncol. (2018) 36 (15_suppl):6009. 10.1200/JCO.2018.36.15_suppl.6009 PMC8462641 32822275

